# Rapid Fabrication of Superhydrophobic Virtual Walls for Microfluidic Gas Extraction and Sensing

**DOI:** 10.3390/mi12050514

**Published:** 2021-05-02

**Authors:** Wojciech Raj, Daisy Yang, Craig Priest

**Affiliations:** 1 Institute of Polymer and Dye Technology, Lodz University of Technology, Stefanowskiego 12/16, 90-924 Lodz, Poland; wojciech.raj@dokt.p.lodz.pl; 2 Future Industries Institute, UniSA STEM, University of South Australia, Mawson Lakes, SA 5095, Australia; daisy.yang@unisa.edu.au; 3 Australian National Fabrication Facility—South Australia, University of South Australia, Mawson Lakes, SA 5095, Australia

**Keywords:** virtual walls, gas extraction, ammonia, Laplace pressure, microfabrication

## Abstract

Based on the virtual walls concept, where fluids are guided by wettability, we demonstrate the application of a gas phase extraction microfluidic chip. Unlike in previous work, the chip is prepared using a simple, rapid, and low-cost fabrication method. Channels were cut into double-sided adhesive tape (280 µm thick) and bonded to hydrophilic glass slides. The tape was selectively made superhydrophobic by ‘dusting’ with hydrophobic silica gel to enhance the wettability contrast at the virtual walls. Finally, the two glass slides were bonded using tape, which acts as a spacer for gas transport from/to the guided liquids. In our example, the virtual walls create a stable liquid–vapor–liquid flow configuration for the extraction of a volatile analyte (ammonia), from one liquid stream to the other through the intermediate vapor phase. The collector stream contained a pH indicator to visualize the mass transport. Quantitative analysis of ammonium hydroxide in the sample stream (<1 mM) was possible using a characteristic onset time, where the first pH change in the collector stream was detected. The effect of gap length, flow rates, and pH of the collector stream on the onset time is demonstrated. Finally, we demonstrate the analysis of ammonium hydroxide in artificial human saliva to show that the virtual walls chip is suitable for extracting volatile analytes from biofluids.

## 1. Introduction

Quantitative analysis of volatile or semi-volatile chemical vapors has numerous applications in chemistry and life sciences, such as for environmental monitoring and clinical diagnosis [1,2,3]. Current analysis techniques for volatile gases mainly focus on either direct contact of the sensing probe with the sample matrix [4,5,6], or making use of a membrane (such as denuders or diffusion scrubbers [3,7]) for separating analytes from the sample. Both techniques have their limitations, such as low selectivity or fouling of the sensing probe or membrane. To overcome those drawbacks, an alternative membraneless technique for the separation of gas analyte from liquid samples has been introduced, based on a virtual wall concept [8], where volatile analyte diffuses from the sample reservoir/stream into the collector reservoir/stream through a gap created with hydrophobic “walls”. Dissolution of the vapor gas into the collector solution for analysis enables effective detection from the complex sample matrix. This design concept has been efficiently utilized in a number of fluidic-based techniques for gas separation/sensing to replace the conventional membrane-based devices [9,10,11,12,13,14].

Patterning hydrophobic regions inside channels typically requires modifying the surface in selective areas before or after aligning, and bonding substrates to form enclosed channels for fluids to pass through [8,15]. A critical issue in all of those developments is often the costly and laboratory-intensive fabrication procedure, which usually requires multi-step, clean room processing. Recent efforts have been devoted to finding alternative, lithography-free approaches for chip fabrication, and procedures with controllable micropatterning, such as 3D printing [16,17,18,19], paper-based microchips [20,21,22] and a range of other low-cost microfabrication techniques [23]. The application of double-sided tape with channel structures (cut with a laser engraver or cutter device; also known as xurography or razor writing) has also been reported in recent years, and can ease the design, fabrication and integration of microchips [24,25,26,27,28,29,30]. Although these methods cannot achieve the resolutions of photolithography, they can reduce the cost and time for microchip fabrication. Compared to laser engraving, xurography offers several advantages [28], such as flexible material choice, safer operation, and lower equipment cost. The deployment of xurography as a manufacturing tool has been validated for many types of microfluidic applications [27,30,31,32], as has recently been reviewed [23].

**Figure 1:**
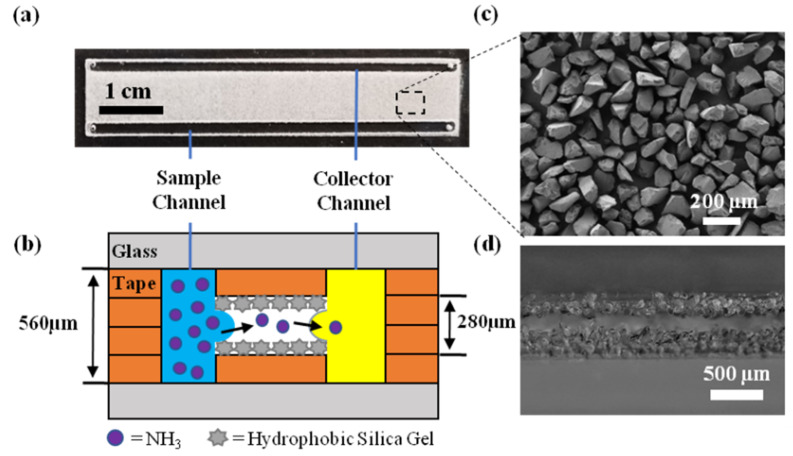
(**a**) Optical image of the microfluidic device with parallel sample and collector channels; (**b**) schematic representation of the mass transfer of analytes from sample stream to collector stream; (**c**) SEM image of the modified glass surface covered by hydrophobic silica gel glued by adhesive tape; and (**d**) microscopy image of the cross-section of the gap.

In the present contribution, we demonstrate the rapid, low-cost, and reliable fabrication of virtual walls in a microfluidic chip, using channels cut into double-sided adhesive tape that is coated with hydrophobic silica gel. By rendering the tape superhydrophobic, the chip can be “open”, with fluids guided by the wettability contrast alone (virtual walls). To demonstrate the operation of these chips, micro-channel geometry was designed to create a liquid–vapor–liquid configuration that allows gas transport from the sample (liquid) to the collector stream (liquid) via the intermediate vapor phase. A schematic representation of gas molecules transported from the sample stream into collector stream is shown in Figure 1. Further details are provided in the chip design section. Unlike previous work, the chip is prepared using a simple, rapid and low-cost fabrication method, providing a new fabrication strategy to create patterned, superhydrophobic surfaces inside channels, for a variety of lab-on-a-chip applications, including gas-diffusion based separation/sensing. For proof-of-concept, ammonia extraction was demonstrated in this device by flowing 5 mM ammonium hydroxide solution through a channel that was parallel to a collector stream of pure water. The extraction of ammonia was visualized by a pH indicator in the collector stream. The effects, such as gap length, flow rate, and pH of the collector stream, were examined for their significant impact on the onset time of ammonia sensing in the collector stream. We show that the microfluidic chip is sensitive to <1 mM concentrations of ammonium hydroxide within a relatively short experimental time (<5 min). Moreover, the detection of mM concentrations of ammonium hydroxide in artificial human saliva was possible, providing the potential for the development of a low-cost ammonia sensor for medical diagnostic applications, or a variety of other gas sensor applications.

## 2. Materials and Methods

### 2.1. Chemicals and Materials

Trichloro(octadecyl)silane (≥90%), silica gel (DavisilTM, grade 644, 100–200 mesh, 150 Å, ≥99%), phenol red (ACS reagent) and sodium chloride (AR), potassium chloride (AR), anhydrous calcium chloride (AR), potassium phosphate monobasic (AR), potassium thiocyanate (AR), sodium sulphate anhydrous (AR), and sodium bicarbonate (AR) were all purchased from Sigma-Aldrich. ARsealTM 90,880 polypropylene double-sided adhesive tape was purchased from Adhesives Research. Cyclohexanes (AR), universal indicator pH 3–11, ammonia 30% solution (AR), and urea were provided by Chem-Supply, Australia. Milli-Q water (18 MΩ·cm resistivity) was used to prepare all the solutions. Different concentrations of ammonium hydroxide solution were freshly prepared and determined using acid-base titration.

### 2.2. Preparation of Hydrophobized Silica Gel

Typically, 2 g silica gel was suspended in 10 mL cyclohexane in a beaker, and then 1 mL trichloro(octadecyl)silane in cyclohexane solution (1 M) was added dropwise to the beaker. The reaction mixture was aged for 30 min at room temperature to form a hydrophobic surface on the silica gel. The modified silica gel was separated by filtration and washed three times with 15 mL cyclohexane. It was then dried in an oven overnight at 60 °C and stored in a sealed vial for further use. 

### 2.3. Preparation of Artificial Saliva

Basic artificial saliva for studying ammonia sensor interference was made by mixing sodium chloride (125.6 mg), potassium chloride (963.9 mg), anhydrous calcium chloride (172.0 mg), potassium phosphate monobasic (654.5 mg), potassium thiocyanate (654.5 mg), sodium sulphate anhydrous (336.5 mg), sodium bicarbonate (630.8 mg) and urea (200 mg) together in 1 L Milli-Q water [33]. Different concentrations of ammonium hydroxide solution were then prepared with this artificial saliva standard solution, and the final pH was adjusted to 11.5 for releasing ammonia gas immediately before the measurements.

### 2.4. Chip Design

There are two designs of the sensor chip. Figure 1a shows the design with parallel channels, for demonstrating the extraction of analytes from the sample stream into the collector stream, through the intermediate gas phase. For quantitative analysis of dissolved ammonia in the collector stream within a convenient (short) experimental time, a new chip was designed with a shorter vapor gap (1.3 mm). Both devices were fabricated by cutting the channel design into double-sided adhesive tape and bonding this to hydrophilic glass slides separated by a spacer. The tape was selectively made superhydrophobic by ‘dusting’ with hydrophobic silica gel in the gap. The gap is used to selectively transfer the vapor (analyte) into the collection (sensing) stream, eliminating interferences from suspended particulates and non-volatile solutes in the sample without using any membranes. The flow rate, pH and gap distance between channels was varied to improve the sensing performance, as discussed later. In this study, ammonia was selected for proof-of-concept. The release of ammonia vapor from the sample solution stream was accelerated by adjusting the pH of the solution to 11.5 using KOH. Sensing of ammonia in the collector stream was performed by spectroscopic detection.

### 2.5. Chip Fabrication

**Figure 2:**
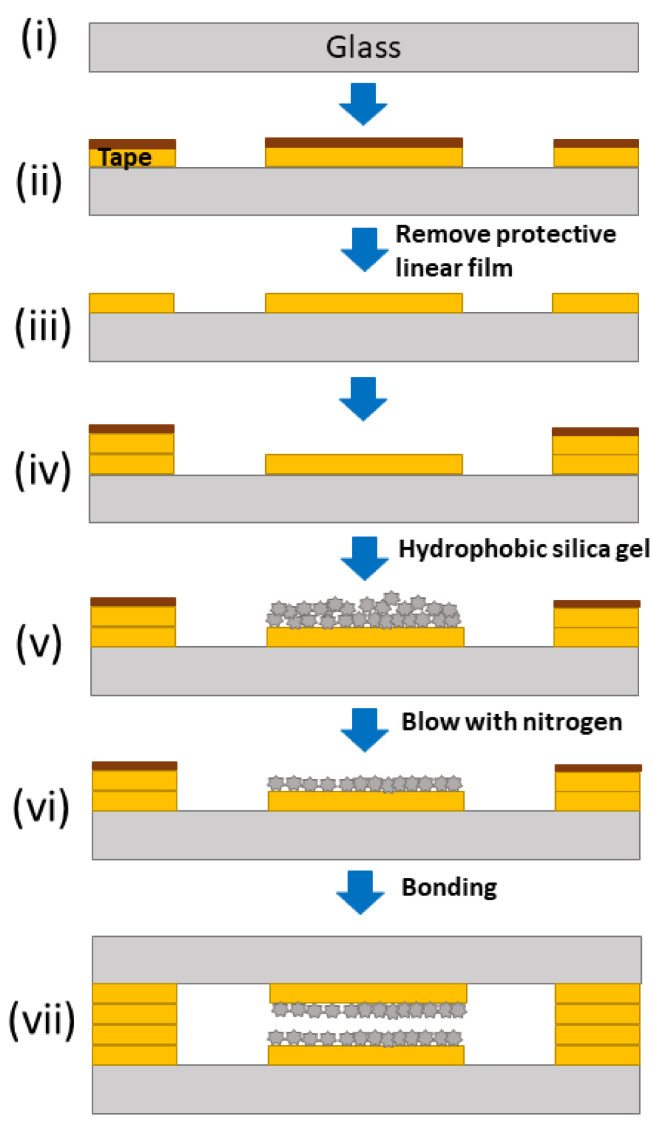
Schematic representation of the microchip fabrication: (i) bare glass substrate, (**ii**) fix adhesive tape with channel design, (**iii**) remove protective liner from adhesive tape, (**iv**) fix adhesive spacer, i.e. around edge of chip, (**v**) add hydrophobic silica gel to exposed adhesive tape, (**vi**) remove excess hydrophobic silica gel, (**vii**) close chip with identical upper plate. See main text for details.

A schematic representation of chip fabrication is presented in Figure 2. The two halves of the chip were prepared in parallel, and then contact bonded by adhesive tape. The details of the fabrication were described as follows: (i) Borosilicate glass (Borofloat^®^ 33, 30 × 70 mm^2^) substrates were cleaned with acetone and isopropyl alcohol to remove any surface contamination; (ii) the designed pattern was cut out in polypropylene double-sided adhesive tape using Silver Bullet Professional Series, and the cut pattern was glued on to the glass substrate; (iii) the protective liner film was then removed from the tape; (iv) another layer of tape was cut and glued to the substrate along the frame of the chip to create an empty space between the two channels; (v) the uncovered empty space between the two channels was then sprinkled with an excess amount of hydrophobic silica gel to ensure a complete coverage, and a thin layer of hydrophobic silica gel was immediately glued onto the substrate by the adhesive tape; (vi) the unglued silica particles were removed by blowing the surface with nitrogen; (vii) the other half of the chip was prepared in the same way on a glass substrate with pre-drilled standard inlet and outlet ports (off-the-shelf component), and the two halves were bonded to each other by removing the protective linear film. For the channel size used in this study, manual alignment was sufficient. An open space with hydrophobic walls was then created between the two channels, which allowed the pass-through of vapor gas but not suspended particulates and non-volatile solutes.

### 2.6. Operating Procedure

For optical observation of ammonia transferring from the sample stream into the collector stream, 5 mM ammonium hydroxide solution (pH adjusted to ~11.5 using KOH) was introduced into the sample channel by syringe pump, at a fixed flow rate of 2 mL/h. While in the collector channel, pure water (pH ~7) containing universal indicator (a mixture of a variety of indicators, pH 3–11, supplied by Chem-supply, 5% *v/v*) was introduced by syringe pump at four different flow rates (0.75, 1, 1.5 and 2 mL/h, respectively). Generally, we allowed two hours for the system to reach a steady state, to ensure reliable measurements; however, later we show that shorter time-scale measurements are possible. The process of the ammonia diffusing into the collector stream was captured by a series of optical images. Video analysis software (Tracker 5.0.6) was used to extract color scale data (i.e., RGB region) from the image files for the determination of the diffusion boundary of the ammonia into the water, which is reflected by the color change of the indicator. Based on the diffusion profile, diffusion coefficients of the ammonia-in-water phase at different flow rates are calculated. For each flow rate at the collector channel, a series of four independent measurements were conducted. 

For concentration-dependent sensing of ammonia on the chip, the operation procedure was similar, but slightly different from above. Ammonium solutions of different concentrations (0.114, 0.228, 0.342, 0.57, 0.798 and 0.912 mM, respectively) with pH adjusted to ~11.5 were introduced into the sample channel by a syringe pump at a fixed flow rate of 2 mL/h or 10 mL/h. At the same time, 0.1 M KCl solution containing 0.3 mM phenol red was introduced into the collector channel by syringe pump. Note that weak acidity (pH = 3.6) was detected in the original collection solution, which was used as prepared and adjusted to pH 4.4 for comparison, and for further ammonium calibration in both standard and artificial saliva samples. The flow in the collector channel was then stopped for the concentrating of ammonia absorption, to ensure effective spectroscopic measurement. A UV-vis spectrometer (Ocean View QE65000 detector) connected to a modified Olympus BH2 microscope was mounted on the top for collecting spectroscopic data from the experiments. This setup enabled the analysis of a 70 × 100 μm^2^ area (i.e., the measuring point, under 5× magnification objective) in the collector channel, close to the diffusion gap (middle). Quantitative analysis of dissolved ammonia in artificial saliva was similar under a condition of 2 mL/h flow rate (sample stream) and pH 4.4 (collection solution) at room temperature. Error bars are provided for each measurement, based on three repeats. Between each measurement, the device was cleaned with water and blown dry with nitrogen. 

### 2.7. Surface Characterization

The distribution of hydrophobic silica gel on the glass substrate, glued by adhesive tape, was characterized by ZEISS SEM Crossbeam 540. The hydrophobicity of the modified glass surface was confirmed by contact angle (CA) measurements. Advancing and receding contact angles were measured using the sessile drop method, based on the observation of the profile of the droplet (Dataphysics OCA-20). Advancing ($\theta_{ad}$) or receding ($\theta_{re}$) CA were measured by sequentially increasing or decreasing the volume of the water droplet deposited on the surface with a small syringe, and were recorded as the contact line began to advance or retract. The CA hysteresis was defined as ($\theta_{ad}\;$−$\;\theta_{re}$). Optical images of the cross-section of the chip were captured using a microscope camera (LEICA-DFC310-FX) mounted to a microscope (LEICA-Z16-APO).

## 3. Results and Discussion

### 3.1. Surface Characterization

**Figure 3:**
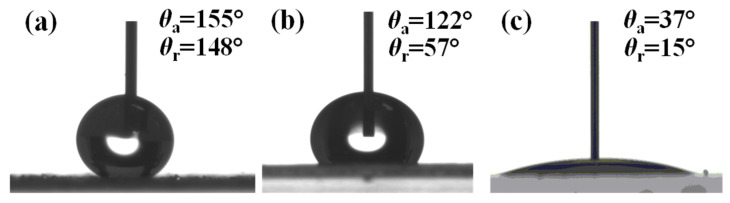
Images showing the advancing water contact angels on: (**a**) polypropylene tape covered with superhydrophobic silica gel; (**b**) polypropylene tape after removing protective linear film; and (**c**) clean glass substrate.

The water contact angles of the clean glass surface, glass/tape surface, and the glass/tape/silica gel surfaces are shown in Figure 3. The clean glass surface was hydrophilic with a water contact angle of 37° ($\theta_{ad}$) and 15° ($\theta_{re}$), respectively. The glass/tape surface (with protective liner film removed) was hydrophobic with a static advancing contact angle of 122° ($\theta_{ad}$), which is similar to that of previous studies on polypropylene surfaces [34,35], but a high contact angle hysteresis (65° with water) was observed on the surface. After coating the adhesive with hydrophobized silica gel (glass/tape/silica gel), the static advancing contact angle increased to 155° ($\theta_{ad}$), and the contact angle hysteresis decreased to 7°. The latter confirmed that the glass/tape/silica gel surface was superhydrophobic and, therefore, suitable for creating virtual walls in the chip. A high-resolution image of the glass/tape/silica gel surface (Figure 1c) shows that the silica gel particles thoroughly cover the tape but also create air gaps between the hydrophobic particles that yield Cassie state wetting [36] and, in this case, super-hydrophobicity.

### 3.2. Flow Stability Consideration

In this study, ammonia diffuses from the sample channel to the collector channel through the vapor gap that separates the two liquids, which are guided by the virtual walls, as illustrated in Figure 1b. To avoid liquids spreading into this gap, the pressure along the full length of the liquid streams must be less than the capillary pressure,$\;\Delta P_{C}=\frac{2\gamma cos\theta }{R}$, at the virtual wall, where γ is the liquid–vapor interfacial tension (72.6 mN/m, measured), θ is the contact angle (155°, measured), and *R* is half the air gap height (140 μm). The pressure in the liquid stream is the sum of the hydrodynamic, $\Delta P_{HD}=\frac{8µLQ}{\pi R_{h}{}^{4}}$, and hydrostatic, $\Delta P_{HS}=\rho gh$, pressures, where µ is the fluid dynamic viscosity (0.89 mPa.s), *L* is the length of the channel (60 mm), *Q* is flow rate (0.75–10.0 mL/h), R_h_ is the hydrodynamic radius (0.196 mm), ρ is fluid density (997 kg/m^3^), *g* is gravitational acceleration (9.81 m/s^2^), and *h* is the vertical height of the fluid above the chip in the inlet/outlet tubing (~20 mm). The condition for stable flow is Equation (1).

(1) $$\Delta P_{HD}+\Delta P_{HS}<\Delta P_{C}$$

Table 1 lists the calculated values of each pressure under the typical flow conditions examined in this study, to ensure that flow is stable during experimental measurements. 

### 3.3. Extraction of Ammonia

**Figure 4:**
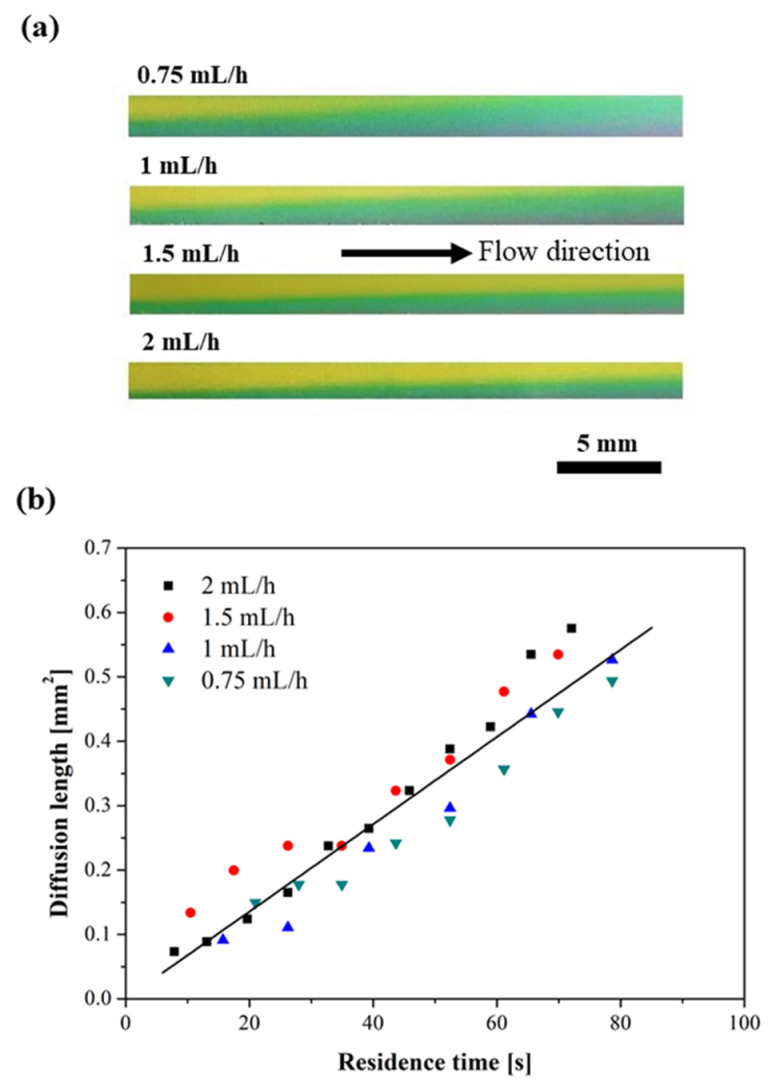
(**a**) Images showing the diffusion of ammonia into the collector stream at four different flow rates; (**b**) plot of the square of diffusion length over residence time along the flow path for four different flow rates (the solid line shown in the figure is the best fit to all of the data).

Figure 4a shows the detection of ammonia in the water stream with universal pH indicator, which provides a clear color change from yellow (pH ~7), to green (pH ~8), and then to purple (pH ~9), along the flow path and at different flow rates, after the liquid–vapor–liquid system reached a steady state. Note that the color of the original water in the collector stream with pH indicator is yellow (pH ~7). Therefore, a color change from yellow to green indicates the absorption and diffusion of ammonia into the collector stream. As more ammonia is absorbed, the indicator turns to a darker color (i.e., purple). It is clearly seen that at a lower flow rate, ammonia diffused further into the channel due to longer residence time. Based on the profile of ammonia diffusing into collector water stream along the flow path, it is possible to extract a series of data (i.e., diffusion length and residence time) for the calculation of the ammonia diffusion coefficient in water (*D*) for each different flow rate.

Along the flow path in the collector channel, the diffusion length of ammonia into water *d* (which is defined as the distance from the vapor-pure water interface (i.e., virtual wall), to the boundary of green–yellow color change) is related to the residence time *t* and diffusion coefficient *D*,

(2) $$d^{2}=Dt$$

where residence time (*t*) is determined by

(3) $$t=\frac{Ax}{Q}$$

where *A* is the cross-sectional area of the collector channel, and *x* is the downstream distance of the measurement location relative to the first exposure to the vapor phase. *Q* is the flow rate of collector stream. In principle, the diffusion length should increase linearly with *t^1/2^*. The linear fit of the experimental data for all different flow rates gives a diffusion coefficient of 6.78 × 10^−5^ cm^2^/s, close to previously reported values [37,38].

### 3.4. Concentration-Dependent Sensing on the Chip

**Figure 5:**
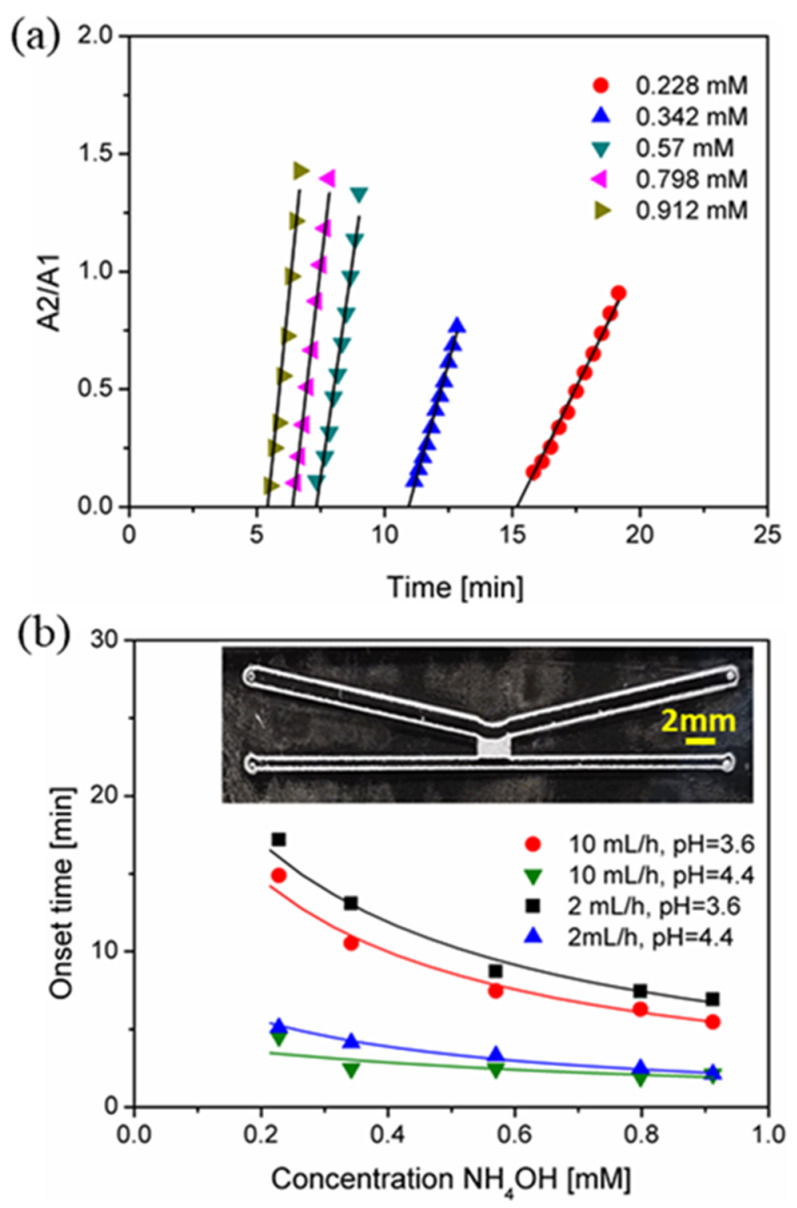
Concentration-dependent ammonia sensing on the chip: (**a**) linear fitting of the ratio of A2/A1 vs. experimental time for determination of onset time at flow rate of 10 mL/h at pH 3.6; and (**b**) onset time versus ammonium concentration at two different flow rates (2 and 10 mL/h in the sample channel) and two different pHs (3.6 and 4.4, respectively).

A modified design (shown in Figure 5b, inset) with shorter vapor diffusion length was used for the quantitative analysis of dissolved ammonia from the sample solution. During the measurements, 0.1 M KCl solution containing phenol red was used as the pH indicator in the collector channel. Phenol red was chosen because of its color change over a narrow pH range (6.0 to 8.5), which falls into the range of pH change in this study. Phenol red has two distinct absorbance bands at 439 nm (A1) and 559 nm (A2) in the visible range, which change dramatically between pH 6 (yellow) and 8 (pink/red), and are separated by an isosbestic point at 479 nm [39]. A1 and A2 refer to the absorbances measured at 439 nm and 559 nm, respectively. In this study, for a given condition (i.e., constant flow rate in the sample stream and no flow in the collector stream), phenol red shows clear change of dominant peak from 439 nm (A1) at 0.1 M KCl (pH 3.6 or 4.4) to 559 nm (A2) due to the absorption of ammonia in the collector stream. The change of the ratio A2/A1 was recorded as a function of time during the ammonia concentrating process in the collector stream. It is noted that there is a linear relationship between the ratio of A2/A1 and the experimental time (or resident time) before it reaches a saturated state. Figure 5a shows a typical example of the linear relationship between the ratio of A2/A1 and the experimental time at a fixed sample flow rate of 10 mL/h, collection solution pH 3.6 and the room temperature, which is also observed for all concentrations of ammonium examined at different conditions in this study. By extrapolating the linear fits, it is possible to determine an onset time at which we first detect a color change and relate this to the concentration of ammonium in the sample stream.

Figure 5b shows that by changing the concentration of ammonium in the sample solution, the proposed microfluidic platform shows a sensitive response (onset time) to increasing concentrations of ammonium in the sample stream. During the measurement, it was confirmed that ammonium concentration, flow rate (sample stream) and pH of the collection solution had significant impact on the onset time of ammonia in the collector stream. Higher ammonium concentration, faster flow rate and higher pH of the collector stream resulted in earlier detection of ammonia. Compared to flow rate, the pH of the collector stream has shown a more predominant effect on ammonia onset time. At pH 4.4, it took around 5 min to observe a color change in the collector stream at the lowest concentration of ammonium (around 0.1 mM) while it took 40–60 min to observe it at pH 3.6 (not shown in the figure).

**Figure 6:**
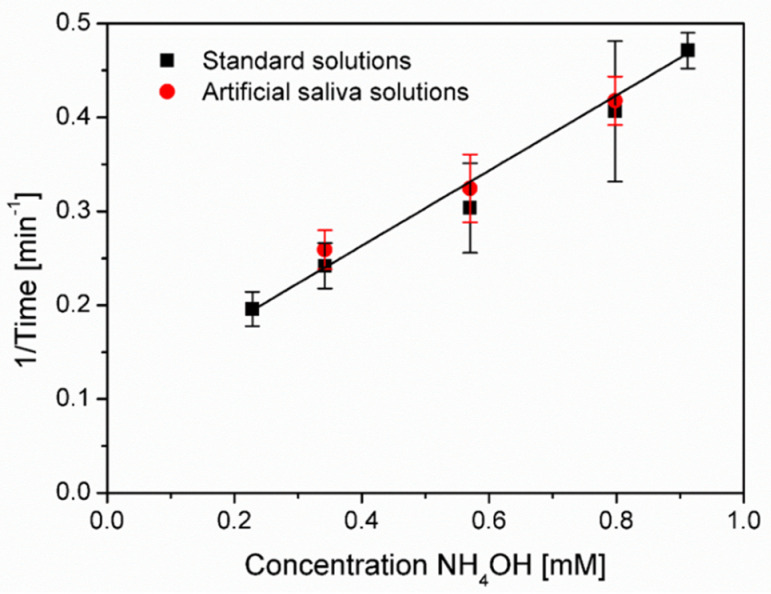
Calibration curve for ammonia sensing in standard and artificial saliva solutions.

A linear calibration relationship (with R^2^ ≈ 0.99) between the reciprocal of onset time (1/*t*) and the concentration of ammonium was determined within the range of 0.2–1 mM under the optimal conditions (pH 4.4 and flow rate 2 mL/h), shown in Figure 6.

### 3.5. Ammonia Sensing in Artificial Saliva

Many real samples contain multiple interferences (e.g., interference ions and suspensions). In this study, an artificial saliva solution containing the mixture of several common salts and urea was prepared to verify the robustness of the proposed microfluidic method, for selective detection of ammonia from interferences. The calibration curve for dissolved ammonia concentration was obtained as shown in Figure 6. The same relationship was observed when using ammonia-spiked artificial saliva as the sample (<1 mM), compared with pure ammonium solutions under the same conditions. It verified the microfluidic method for detecting volatile ammonia gas vapor from the complex sample matrix, providing the potential for medical diagnostic applications, such as detection of some stomach bacterial infection, e.g., *H. pylori* infection, which is a primary identified cause of stomach cancer [40].

## 4. Conclusions

In this study, a novel fabrication strategy was developed for creating superhydrophobic “virtual walls” inside channels, for the extraction and sensing of volatile gas from aqueous samples. Unlike previous work, the chip is prepared using a simple, rapid, and low-cost fabrication method by cutting channel structures into double-sided adhesive tape and bonding this to hydrophilic glass slides. The separation of analytes from the sample solution was realized by virtual walls, where a liquid–vapor–liquid system was guided by selective modification of the tape with hydrophobized silica gel. It has been demonstrated that the use of the microfluidic chip, combined with spectroscopic detection, offers sensitive detection of dissolved ammonia from water phase, including complex aqueous samples (e.g., artificial saliva solution). This has provided the potential for developing low-cost ammonia sensors for medical diagnostic applications, or a variety of other lab-on-a-chip applications where controllable surface patterning is required. 

## Figures and Tables

**Table 1:** Calculated pressures at different flow rates (assuming a standing tubing height of 2 cm).

Flow Rate, mL/h	Liquid Pressure, [Pa]			Resistant Pressure, [Pa]
	$\Delta P_{HD}$	$\Delta P_{HS}$	$\mathrm{Sum}\;(\Delta P_{HD}+\Delta P_{HS})$	Capillary Pressure
0.75	19	196	215	470
1	26	196	221	470
1.5	38	196	234	470
2	51	196	247	470
10	256	196	452	470
